# Comparing the effectiveness of selective laser trabeculoplasty with topical medication as initial treatment (the Glaucoma Initial Treatment Study): study protocol for a randomised controlled trial

**DOI:** 10.1186/s13063-015-0924-6

**Published:** 2015-09-11

**Authors:** Ecosse L. Lamoureux, Rachel Mcintosh, Marios Constantinou, Eva K. Fenwick, Jing Xie, Robert Casson, Eric Finkelstein, Ivan Goldberg, Paul Healey, Ravi Thomas, Ghee Soon Ang, Konrad Pesudovs, Jonathan Crowston

**Affiliations:** Centre for Eye Research Australia, University of Melbourne, the Royal Victorian Eye and Ear Hospital, Melbourne, 3002 Australia; Singapore Eye Research Institute, Singapore National Eye Centre, Singapore, Singapore; Duke, Graduate Medical School, Singapore, Singapore; University of Adelaide, North Terrace, Adelaide, South Australia Australia; Discipline of Ophthalmology, University of Sydney, Sydney, New South Wales Australia; Glaucoma Unit, Sydney Eye Hospital, Sydney, New South Wales Australia; Eye Associates, Sydney, New South Wales Australia; Centre for Vision Research, Department of Ophthalmology and Westmead Millennium Institute, University of Sydney, Sydney, NSW Australia; Queensland Eye Institute, 149 Melbourne Street, South Brisbane, Queensland Australia; University of Queensland, Brisbane, Queensland Australia; Royal Victorian Eye and Ear Hospital, Victoria, Australia; NH&MRC Centre for Clinical Eye Research, Discipline of Optometry and Vision Science, Flinders University and Flinders Medical Centre-Australia, Adelaide, Australia

**Keywords:** Glaucoma, eye drops, selective laser trabeculoplasty, quality of life, cost-effectiveness, randomised clinical trial

## Abstract

**Background:**

Glaucoma is the leading cause of irreversible blindness in the world. Estimated to affect 60 million people worldwide, this figure is expected to rise to 80 million by 2020. Untreated, glaucoma leads to visual decay and eventually to blindness, and can significantly reduce quality of life. First-line treatment in patients with primary open-angle glaucoma and exfoliative glaucoma is topical medical therapy with ocular hypotensives as eye drops. However, eye drops have several disadvantages including cost, possible local and systemic side effects, and adherence and perseverance issues. Randomised controlled trials have demonstrated that selective laser trabeculoplasty is equally as effective in lowering intraocular pressure as eye drops. However, the impact of these two treatment modalities from the patient and economic perspectives has not been adequately determined. Thus, it remains unclear whether topical medical therapy or selective laser trabeculoplasty should be recommended as first-line treatment for glaucoma.

**Methods/Design:**

This protocol describes an international, multi-centre, randomised controlled trial to determine the optimum first-line therapy for people with primary open-angle glaucoma and exfoliative glaucoma. This study will compare the effect of selective laser trabeculoplasty and topical medication with respect to patients’ generic and glaucoma-specific quality of life. The trial will also provide a detailed cost-effectiveness analysis and compare the clinical effectiveness with respect to the degree of intraocular pressure lowering and rates of treatment failure. Research coordinators in each centre will identify and recruit previously untreated patients with primary open-angle glaucoma and exfoliative glaucoma. Those who meet the eligibility criteria will be invited to enter a randomised controlled trial with either selective laser trabeculoplasty or topical ocular hypotensive therapy, according to a stepped regimen. Outcome assessment will be measured at 6 weeks and at 6, 12, and 24 months post-treatment. Regular clinic follow-ups will continue as clinically indicated between study outcome visits.

**Discussion:**

The Glaucoma Initial Treatment Study is the first multi-centred RCT to determine the optimum first-line therapy for people with glaucoma. Our trial will have an unprecedented capacity to meaningfully transform the treatment and management of glaucoma in Australia and overseas.

**Trial registration:**

ACTRN12611000720910; Date registered: 11 July 2011

## Background

Glaucoma is the leading cause of irreversible blindness in the world [1]. Estimated to affect 60 million people worldwide, this figure is expected to rise to 80 million by 2020 [2]. Glaucoma represents a group of optic neuropathies characterised by a progressive degeneration of retinal ganglion cells and their axons resulting in structural changes of the optic nerve and retinal nerve fibre layer with concomitant patterns of visual loss. Untreated, glaucoma leads to visual decay and eventually to blindness, although less severe glaucoma can also significantly reduce quality of life (QoL) and cause economic burden to both the individual and society [3–5].

The pathophysiology and factors contributing to the onset and progression of glaucoma are not fully understood. Reduction in intraocular pressure (IOP) is the only modifiable risk factor known to delay glaucoma onset and progression [6]. IOP is regulated by a balance between the rates of aqueous humour secretion by the ciliary body epithelium and its drainage via the trabecular meshwork and uveoscleral outflow pathways. First-line treatment in patients with glaucoma is topical medical therapy with ocular hypotensives as eye drops. These either increase the aqueous outflow (such as prostaglandin analogues) or reduce aqueous production (such as beta-adrenergic antagonists). Topical medical therapy effectively lowers the IOP [6] but has limitations and disadvantages including cost, possible local and systemic side effects, adherence and perseverance issues, physical barriers to self-instillation (such as tremor or arthritis) and the prospect of daily and often life-long commitment to medications [7–21].

The use of laser trabeculoplasty to lower IOP has provided an alternative strategy to treat glaucoma. One of the laser trabeculoplasty modalities is selective laser trabeculoplasty (SLT), which utilises a Q-switched, frequency-doubled Nd:YAG (532 nm) laser. It lowers IOP in patients with glaucoma by enhancing aqueous outflow through the trabecular meshwork, but the exact mechanism underlying the effectiveness of SLT remains uncertain. SLT has been shown to recruit monocytes to the trabecular meshwork and induce disassembly of tight junctions in Schlemm’s canal cells [22, 23]. In comparison with older laser trabeculoplasty techniques, SLT induces less coagulative damage and structural change to the meshwork and is potentially repeatable [24]. However, the effect of SLT diminishes over time and it may be associated with local adverse effects such as post-laser inflammation and temporary elevations in IOP.

Studies comparing these treatments have demonstrated no significant differences between prostaglandin eye drops and SLT in lowering IOP [25, 26], although one study showed that the prostaglandin analogue latanoprost controlled IOP fluctuations better than SLT [27]. From the societal perspective, a published cost-analysis found SLT to be less costly than most brand-name topical medications within 1 year and less costly than generic latanoprost and generic timolol (beta-adrenergic antagonist) after 13 and 40 months, respectively [28].

Although these studies have indicated that SLT and topical prostaglandins are equally effective in lowering IOP, they were generally underpowered and did not consider the impact of these two treatment modalities from the patient and economic perspectives. Glaucoma patients can experience reduced QoL due to associated functional loss; emotional and social implications; and the inconvenience, side effects and cost of treatment [3, 4, 29–34]. Thus, it remains unclear whether topical medical therapy or SLT should be recommended as first-line treatment for glaucoma with corresponding improvements in QoL and economic benefits.

### Objectives and hypotheses

We are implementing a multi-centre randomised controlled trial (RCT) to determine the optimal first-line therapy for people with primary open-angle glaucoma (POAG) and exfoliative glaucoma (XFG). Our primary endpoint will be health-related QoL (HRQOL). This study will evaluate treatment outcomes with respect to patient QoL, provide a detailed economic effectiveness analysis, and compare the clinical effectiveness in terms of the degree of IOP lowering and rates of treatment failure.

We hypothesise that treatment with SLT, compared with topical medication, will improve overall and glaucoma-specific QoL parameters, show comparable IOP reduction and demonstrate less ocular and systemic side effects, including less frequent ocular surface disease. We postulate that SLT treatment is more cost-effective than topical medical therapy in the treatment of POAG and XFG.

### Specific aims

Aim 1: Our first aim is to compare the effect of SLT and topical medication on glaucoma-specific QoL at 6 weeks and at 6, 12, and 24 months post-treatment (primary outcome).

Aim 2: Our second aim is to quantify the cost-effectiveness of SLT treatment compared with glaucoma medication treatment.

Aim 3: Our third aim is to determine factors associated with treatment outcomes at 12 and 24 months post-SLT, including the rate of IOP success, defined as >25 % reduction in IOP from baseline measurements.

Aim 4: Our final aim is to assess the impact of SLT and eye drops on safety, laser-linked complications, and common side effects for glaucoma patients after the initiation of treatment.

## Methods/Design

The Glaucoma Initial Treatment Study (GITS) is a multi-centred, cluster randomised, controlled, clinical trial comparing two treatments for patients with POAG and XFG. Treating investigators and patients are not masked to treatment allocation due to the nature of the treatments; however, the QoL questionnaire administrators are masked to treatment allocation.

### Inclusion and exclusion criteria

#### Inclusion criteria

Inclusion criteria are as follows:

1. 35 years of age and above.
2. Previously untreated patients with either POAG or XFG that, in the investigator’s opinion, warrants IOP-lowering treatment.
3. Visual field - mean deviation (MD) values between 0 and -12 dB at baseline in the study eye on the Humphrey Visual Field Analyser.
4. Optic disc changes consistent with glaucoma including rim loss, nerve fibre layer defects (NFLD), and disc haemorrhages.

#### Exclusion criteria

1. History or evidence of glaucoma other than POAG or XFG.
2. Advanced glaucomatous field loss with MD > -12 dB.
3. History of use of topical or systemic ocular hypotensive medication(s).
4. Previous intraocular surgery (including glaucoma laser or glaucoma surgery), with the exception of uncomplicated phacoemulsification that did not require additional intervention for complications.
5. Iridotrabecular drainage angle anomalies.
6. Evidence of moderate non-proliferative diabetic retinopathy or worse, neovascularisation or rubeosis iridis.
7. Current use of a systemic corticosteroid, epinephrine or clonidine.
8. Patients, who in the opinion of the investigator, are at high risk of suffering symptomatic vision loss and/or may require glaucoma surgery within the 2-year follow-up to the study.
9. Patients who are pregnant or currently breastfeeding and/or planning to become pregnant within the study period.
10. Patients who have a condition or are in a situation, which, in the investigator’s opinion may put them at significant risk or may interfere significantly with the patient’s participation in the study.

Written informed consent will be collected from each participant prior to inclusion in the study.

### Interventions

**Fig. 1:**
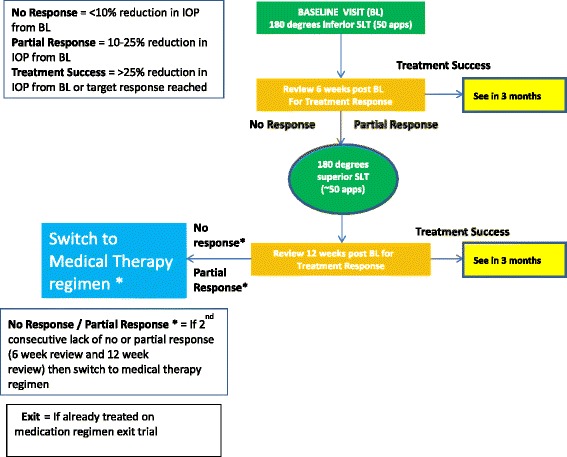
Selective laser trabeculoplasty (SLT) treatment regimen: participants will follow a stepped treatment regimen, depending on their response to treatment. App=applications; BL=baseline; IOP=intraocular pressure; SLT=selective laser trabeculoplasty

**Fig. 2:**
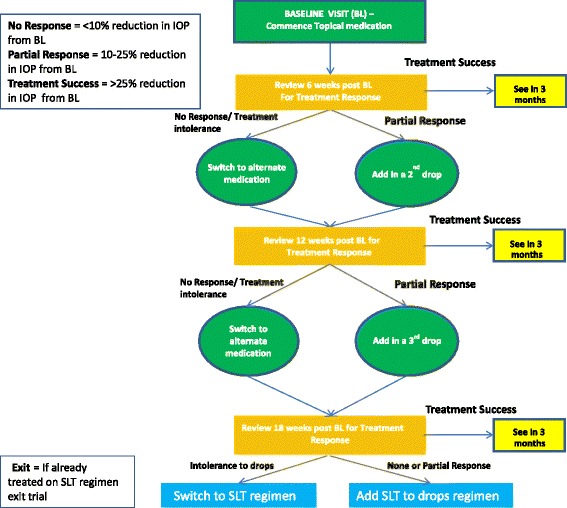
Topical medication treatment regimen: participants will follow a stepped treatment regimen, depending on their response to treatment. BL=baseline; IOP=intraocular pressure; SLT=selective laser trabeculoplasty

Patients will be randomised 1:1 to treatment with either SLT or topical ocular hypotensive therapy, according to a stepped regimen (Figs. 1 and 2). If both eyes meet the inclusion criteria, the eye with the highest IOP will be enrolled in the study. However, both eyes will receive the same treatment initially. If both eyes have the same level of IOP, it will be at the investigator’s discretion to choose the study eye based on visual fields and optic disc evaluation.

### Selective laser trabeculoplasty

For patients assigned to the SLT treatment arm, a single application of pilocarpine (1 %) and apraclonidine (1 %) or brimonidine (0.2 % or 0.15 %) will be instilled into the operative eye prior to the laser (Fig. 1). A frequency doubled, q-switched Nd:YAG laser emitting at 532 nm with a pulse duration of 3 ns, a spot size of 400 μm, and pulse energies ranging from 0.2 to 1.7 mJ, coupled to a slit lamp delivery system with a helium-neon laser aiming system will be used in all cases. Any mirrored gonioscopic lens that does not magnify is acceptable for use during the procedure. The laser will be focused on the trabecular meshwork using the HeNe aiming beams and a single laser pulse will be delivered at the 3 o’clock position. The laser energy will be increased or reduced by 0.1-mJ increments until fine ‘champagne’ bubbles are generated. During laser application, bubble formation will be monitored with each pulse to avoid excessive bubble formation and to avoid no observed response.

Initial 180° treatments of approximately 50 applications will be applied inferiorly from the 3 to 9 o’clock position. The total number of pulses delivered and the total amount of energy delivered will be recorded following each treatment. Participants will return for review of treatment response in 6 weeks from the baseline treatment and will then follow a stepped regimen, depending on their response to treatment. If a second SLT treatment is required, it will be applied over 180° from the 9 to 3 o’clock positions (superiorly). Treatment changes may also be initiated if there is an adverse event post-treatment and if this is considered severe enough to warrant a change in treatment.

### Topical ocular hypotensive therapy

Patients randomised to the topical medication treatment group will be initially prescribed, where possible, a prostaglandin analogue with one drop to be instilled in each eye (or the study eye only if only one eye is enrolled) once daily (Fig. 2). We will instruct patients on how and when to instil their drops and stress the importance of adherence. Patients will return for review of treatment response 6 weeks from the baseline initiation of treatment and will follow a stepped regimen, depending on their response. We will change treatment if there is an adverse event that is considered severe enough to warrant such change or if there is progression in visual field loss or optic disc changes. Treatment response will be defined as follows:

1. Successful response ≥25 % IOP reduction compared with baselines.
2. Partial response = 10-25 % IOP reduction compared with baselines.
3. No response = ≤10 % IOP reduction compared with baselines.

If patients have crossed over with both treatment regimens, they will exit the trial with further management at the discretion of the treating doctor (Figs. 1 and 2).

Based on the grading system developed by the Collaborative Initial Glaucoma Treatment Study (CIGTS) [35], optic discs will be clinically analysed for the following characteristics: (a) vertical and horizontal central cup to disc ratio; (b) focal and diffuse thinning of neuro-retinal rim; (c) presence of a disc haemorrhage; (d) nerve fibre layer defects; and (e) extent of peripapillary atrophy. If a change in treatment is deemed necessary and urgent, it may be initiated by the site investigator although the images would be reviewed by the Optic Disc Outcomes Committee (ODOC), masked to the treatment allocation (and initially the temporal sequence of the photos) to validate treatment change. For all disc changes considered non-urgent, these images will be referred to the ODOC where it will be the final arbiter on the presence/absence of optic disc changes and any alteration to treatment regimens. Visual field progression will be defined according to the following criteria identified by the Glaucoma Progression Alert on the Humphrey Visual Field result:

1. Possible progression = 3 or more points show deterioration on at least 2 consecutive visits.
2. Likely Progression = 3 or more points show deterioration on at least 3 consecutive visits.
3. No progression detected = none of the above conditions is met.

At each visit, the patient’s visual field results will be reviewed and evidence of progression will be initially determined by the Guided Progression Analysis (GPA) alert and the site investigator. If the alert message is that of ‘Possible Progression’, then the visual fields will be forwarded to the Visual Field Outcomes Committee (VFOC), masked to the treatment administered to the patient. This committee will make the final determination on the presence/absence of progression and change to treatment regimens. If, however, the alert message is that of ‘Likely progression’, the investigator is permitted to change/alter treatment and then refer the fields to the VFOC to confirm this progression.

### Randomisation

Randomisation schedules developed by the Centre for Eye Research Australia (CERA) Melbourne, (Coordinating Centre), using a list of computer generated pseudo-random numbers are designed to yield an assignment ratio of 1:1. Randomisation, per person, will be stratified by clinical centre and type of glaucoma. The randomization number will be assigned to a patient sequentially according to the order of enrolment within the stratum. The treatment assignment form, contained in an opaque envelope will be filed in the patient’s ‘Unmasked study folder’ with the patient’s screening code completed on this form. Sites will then scan a copy of the treatment assignment and email this to the Coordinating Centre for its records. When the number of envelopes on site reaches only four remaining, the site will notify the Coordinating Centre, which will send more envelopes containing treatment allocations.

### Allocation concealment

Each study site will receive a block of sequentially numbered, sealed, and opaque envelopes containing treatment allocation for either ‘SLT’ or ‘Drops’. Opaque envelopes will be opened in order of sequence per site. A unique study code will be assigned to each patient at randomisation, consisting of a three-digit randomisation number along with the patient’s initials.

### Outcome measures

#### Health-related QoL (HRQOL)

Considering (a) the substantial side effects of topical medication(s) and poor adherence and (b) its considerable impact on daily living and QoL, HRQoL parameters are our critical outcomes to determine the treatment of choice for glaucoma. The *primary patient-centred outcome* will be assessed by significant changes in two glaucoma-specific scales and one generic HRQoL instrument at 6 weeks and at 6, 12, and 24 months post-treatment. These tools include the following:

1. *Glaucoma Outcome Assessment Tool (GOAT):* We have developed a glaucoma-specific QoL item bank, GOAT, which contains 342 items across 10 specific QoL domains [36]. An item bank is a pool of calibrated items that measure a defined underlying trait such as QoL and becomes operational by the use of computer adaptive testing [37].
2. *The Glau-QoL questionnaire:* The Glau-QoL is a comprehensive glaucoma-specific QoL instrument and has been validated in patients with glaucoma [38].
3. *The AQoL-7D:* The AQoL-7D is a health-related multi-attribute utility instrument to assess QoL [39, 40]. This descriptive system comprises seven domains describing QoL (including vision) with levels of increasing severity. A scoring algorithm, based on general population values for health states defined by the descriptive system, is used to assign utilities to each health state (domain) described by the instrument and overall QoL. The AQoL-7D has been used in several RCTs in Australia and its overall and subscale measures have been found to be sensitive to interventions [41–44]. All QoL outcome measures will be interviewer-administered.

#### Clinical

Clinical secondary outcomes will be determined by the success rates of IOP reduction (defined as >25 % fall in IOP from baseline), changes in the visual field and optic disc from baseline to 24 months. The times required to reach these changes will be reported. Changes in the optic nerve head will be based on rim thinning, haemorrhage, or an increase in cup to disc (C/D) ratio and visual field changes based on progression.

### Statistical analysis plan

All data analyses will be conducted according to a pre-specified analytical plan using Stata version 12.1.0 (Stata Corp, College Station, TX). All hypothesis testing will be performed at the 5 % two-sided significance level. Analyses and data summaries will be carried out using the intention to treat (ITT) population. The ITT population is defined as all patients registered for active follow-up regardless of non-adherence with the intervention. All patients will be analysed according to the intervention that they were randomised to. A per-protocol analysis will be considered if there are a considerable number of protocol violators.

#### Baseline characteristics

Analyses will be performed for all variables at baseline to detect potential bias in recruitment. Continuous variables will be reported using means ± standard deviations (SD) or median (interquartile range (IQR)). For dichotomous/categorical variables, absolute numbers and percentages will be computed, together with their 95 % confidence intervals (CI). The comparison of means will be carried out using Student’s t test, the Mann-Whitney test, analysis of variance or the Kruskall-Wallis test as appropriate. The difference in proportions between the two treatment groups will be carried out using Chi-square statistics. These comparisons will be made by assessing the prognostic relevance of the difference observed, not through hypothesis testing. Baseline summaries with respect to the main covariates will be presented and discussed from a clinical point of view, irrespective of whether a statistical test indicated a ‘statistically significant difference’ between treatment groups. If we find a strong baseline imbalance in a variable, we will include this variable as a covariate in a sensitivity analysis to allow assessment of the robustness of the conclusions drawn from the primary analysis.

#### Primary analysis

The primary analysis will be ITT analysis, which includes all randomised patients in the groups to which they were randomly assigned, regardless of their adherence with the entry criteria, the treatment they actually received, and of subsequent withdrawal from treatment or deviation from the protocol. The ITT analysis will include all patients with 24-month GOAT, Glau-QoL, and AQoL-7D data. The GOAT will be our main primary outcome. The GOAT and Glau-QoL data will initially undergo Rasch Analysis (a form of Item Response Theory - IRT) using Winsteps software (version 3.90), Chicago, Illinois [45].

We will examine the missing data mechanism (probability distribution of missingness) to determine whether it is missing completely at random (MCAR), missing at random (MCR) or missing not at random (NMAR). Since patient-centred measurements will recur on the same patients, longitudinal HRQoL data will be analysed using multivariate generalised estimating equation (GEE) or generalized linear mixed models (GLMMs). We will adjust for pre-defined variables that we consider are associated with the primary outcome (HRQoL) from the literature. These include clinical variables (for example, baseline glaucoma severity, visual field, visual acuity, optic disc changes, duration of glaucoma), and sociodemographic variables (for example, age, gender). Separate regressions will be conducted for each QoL parameter. These models take into account the correlation within observations on the same subject and allow for inclusion of data on subjects who have only partial follow-up without imputing missing data. To handle multiplicity in the multiple regression models, Bonferroni adjustment will be used.

#### Economic analysis

The purpose of the economic evaluation is to examine the incremental cost-effectiveness of SLT relative to medical care. Costs will be quantified from the health system perspective using an activity based costing approach. Sunk costs will be excluded from the analysis. Effectiveness will be defined in terms of the AQoL-7D utilities. Once costs and effectiveness are known for each intervention arm at both baseline and follow-up, the approach for quantifying cost-effectiveness will follow that described in the literature [46]. As is common practice, incremental cost-effectiveness ratios will be compared with common thresholds adopted by National Institute for Health and Care Excellence (NICE) and World Health Organisation (WHO) for what represents good value for money. In addition to quantifying this ratio, we will also conduct one-way and n-way sensitivity analyses and produce cost effectiveness acceptability curves (CEAC) to show the probability that SLT is cost-effective for a range of monetary values that a decision-maker might be willing to pay for a unit change in quality adjusted life year (QALYs). Finally, because much of the costs of SLT are one-time fixed costs, and topical medication(s) is a recurring cost, if the results show SLT to be at least as effective as topical medication, the average time it takes for SLT to become a dominant strategy will also be quantified. All analyses will be conducted using TreeAge software.

#### Clinical efficacy

To assess clinical efficacy (IOP reduction success), Chi square tests will be used and multivariable logistic regression models with odds ratios (OR) and 95 % CI will be built. Other analyses will be tailored to the nature of the secondary outcome data. Numerical data (for example, IOP and visual acuity) and dichotomous data (presence/absence) will be analysed by the GEE. Survival data (for example, time-to-VF (visual field) progression, IOP becoming uncontrolled again) will be analysed using survival analysis. Stepwise multiple regression analyses for secondary outcomes will evaluate a similar set of potential explanatory factors (covariates) in addition to treatment assignment and those evaluated for the primary analysis. Time-varying covariates will be used for analyses evaluating repeated measures of the outcome, if appropriate. Standard errors of estimates from log-linear models will take account of possible over-dispersion with respect to the assumed models.

To determine the impact of SLT (single or multiple applications) on safety, surgical complications, and common side effects associated with glaucoma treatment, Chi square tests will be used initially and regression models with ORs and 95 % CI will be built. Where appropriate, Poisson regression will be used to compare event rates (for example, adverse events). To determine the predictors of IOP reduction success, survival curves will be constructed using the Kaplan-Meier method. Cox proportional hazards method will be used to adjust for confounders. Hazard ratios (HR) with 95 % CI will be provided to estimate the relative risk.

### Planned subgroup analyses

Analyses for the primary (HRQoL) and secondary outcomes (clinical efficacy) will be stratified for baseline types of glaucoma (POAG and XFG).

### Sample size estimation

The sample size for the study is based on the primary outcome measure of HRQoL at 24 months post-treatment. Our hypothesis is that compared with topical medication, SLT treatment will have a significantly greater positive impact on HRQoL. Mean and standard deviations for the pilot data of GOAT and previous work with the Glau-QoL were used [36, 38]. To detect a 0.156 Logit difference (effect size of 0.3) on the Rasch-analysed GOAT and Glau-QoL at 80 % power with a 5 % level of significance, 175 patients will be needed in each group. Similarly, the mean and standard deviation values for the AQoL-7D were obtained from the literature [39, 47]. To detect a 0.7 difference (effect size of 0.3) on the AQoL-7D between the two groups at 24 months with 80 % power at 5 % level of significance; 176 patients will be needed for each group. Assuming an attrition rate of 15 % after 2 years, 193 patients will be enrolled in each group at baseline. Furthermore, for Aim 3, the study will also have 79 % power to detect a 10 % difference in ‘success rate’ of IOP (defined as ≥25 % reduction in baseline IOP) in the SLT group compared with topical medication using a two-sided log-rank test at the 5 % significance level.

### Side-effects reporting and quantification

Information about transient ocular discomfort and pain; mild uveitis; IOP spikes; the presence/absence and severity of ocular surface disease (OSD) signs and symptoms; anterior chamber reaction (cells and flare 1 hour post-laser) and any other sight threatening and non-sight threatening adverse events will be collected. IOP spikes are defined as an elevation >30 mmHg or >30 % increase within the first 4 weeks post-treatment. The presence or absence of OSD signs and symptoms will be obtained from the results of the biomicroscopy, in particular, concerning whether any hyperaemia and/or superficial punctate epitheliopathy is present and the extent of this and also from the responses provided by participants to the OSD Index Questionnaire. This questionnaire was developed by Allergan, Inc’s Outcomes Research Group to provide a rapid assessment of the severity of symptoms of OSD and their impact on vision-related function and has been validated in English speaking subjects [48]. We obtained permission to use this questionnaire.

An adverse event (AE) is defined as any untoward medical occurrence in a patient administered therapy that does not necessarily have a causal relationship with this treatment. An AE can therefore also be any unfavourable and unintended sign (including an abnormal laboratory finding), symptom, or disease temporally associated with the use of a therapy, whether or not it is related to the therapy itself. Any AE will be recorded on the appropriate case report form. It will be graded by an Investigator at each site for severity and relationship to study treatment. An AE reporting system will be implemented to ascertain the occurrence of both anticipated and unanticipated AEs. When indicated, patients suffering AEs will be referred for appropriate ophthalmic or medical care. Information regarding AEs (including incidence, duration, seriousness, severity, relationship to treatment, and action taken) will be recorded throughout the 2 years of the study. If AEs occur, the first concern will be the study participant’s safety. To determine the relationship (if any) between an AE and the study intervention, a causal relationship is deemed present if a determination is made that there is a reasonable possibility that the AE may have been caused by the intervention.

Safety data will be compiled every 6 months by the unmasked investigator and coordinator and submitted to the Study Project Manager, who will, in turn, forward it to the Data Safety Monitoring Committee (DSMC) for review at 6-month intervals. All AEs that are therapy related and unexpected will also be reported to the site’s Human Research Ethics Committee (HREC).

A serious AE (SAE) will be defined as any AE that results in any of the following outcomes: death, a life-threatening AE, inpatient hospitalization or prolongation of existing hospitalization, a persistent or significant disability/incapacity, or a congenital anomaly/birth defect. Important medical events that may not result in death, be life threatening, or require hospitalization may be considered SAE events when, based upon appropriate medical judgment, they may jeopardise the patient and may require medical or surgical intervention to prevent one of the outcomes listed in this definition. Any SAE occurring during the study period and for at least 28 days after the last administration of therapy will be reported as soon as possible to the Study Project manager and also recorded on the appropriate case report forms. All participants with a SAE will be followed up and the outcomes reported. For other SAEs, the governing HREC will be notified as required by their regulations. The investigator will supply the Project Manager and the HREC with any additional requested information (for example, autopsy reports and terminal medical reports).

### Ethical issues

#### Ethics committee approval

This study has been approved by the HREC of the Royal Victorian Eye and Ear Hospital (Reference number 11/1024H), Westmead Hospital (Reference number 2011/10/4.1 (3391) AU RED HREC/11/WMEAD/229), the Royal Australian and New Zealand College of Ophthalmologists (Reference number 34.11), the Health and Disability Ethics Committees of New Zealand (Reference number 12/STH/13), the Southern Adelaide Clinical Human Research Ethics Committee (Reference number 81.13) and National Research Ethics Services Cambridge East (Reference number 13/EE/0204) the Australian College of Optometry Human Research Ethics Committee (Ref H13 002).

## Discussion

Glaucoma has a substantial impact on QoL, with patients experiencing difficulty participating in daily activities; and poor emotional and social well-being. In addition, there are issues associated with the inconvenience, side effects, and costs of treatment [3, 4, 29–34]. At present, it remains unclear whether topical medical therapy or SLT should be recommended as first-line treatment for glaucoma from patient-centred and economic perspectives. We hypothesise that SLT treatment will demonstrate fewer ocular and systemic side effects and is more cost-effective than topical medical therapy in the treatment of POAG and XFG. We will determine this through a multi-centre RCT.

Because our eligibility criteria stipulate that patients must have previously untreated glaucoma, we recognise that we may have difficulty meeting our recruitment targets at a single site. Moreover, the coordinating site, namely the Royal Victorian Eye and Ear Hospital, is a tertiary eye hospital which tends to treat patients with advanced glaucoma with well-established treatment regimens. Therefore, we are collaborating with multiple sites, both nationally and internationally, to ensure that we reach our desired sample size. This will require site-specific ethics approval from all additional recruitment sites and we are aware that this will increase the amount of time required to set-up and commence the project.

Given that our primary outcome is HRQOL which is assessed by three patient-reported outcome measures, the interview schedule is likely to be quite intensive for participants. In order to reduce participant burden and avoid fatigue, which may compromise data quality, we will ensure participants are offered regular breaks and the required time to complete the interview. It is also possible that participants may drop out of the study due to the high level of participation required, which is a threat to external validity. Therefore, all recruiters, research assistants, and study coordinators have been trained in techniques to optimise retention rates. These include communication strategies to emphasise the benefits of participation, neutralise ambivalence, and respond to patient-centred needs; providing reimbursement for travel expenses; being flexible in assessment times; developing rapport with participants and family members; and maintaining a good tracking system to monitor follow up throughout the study period [49, 50].

This is the first multi-centred RCT to address the issues that have plagued the traditional management of glaucoma using topical medication, and it has the potential to transform our current approach to glaucoma management. The outcome of this trial will also have a significant economic impact on the treatment of glaucoma considering the considerable annual cost of glaucoma in Australia [51]. If SLT proves to be cost effective, multi-billion dollar savings could be achieved. Our trial will therefore have an unprecedented capacity to meaningfully change and transform the treatment and management of glaucoma in Australia and overseas.

## Trial status

The GITS trial started January 2013 and is expected to finish December 2016.

## Abbreviations

AE: adverse event
AQOL-7D: Assessment of Quality of Life - 7 Dimensions
CERA: Centre for Eye Research Australia
CI: confidence interval
CEAC: cost effectiveness acceptability curves
CIGTS: Collaborative Initial Glaucoma Treatment Study
DSMC: Data Safety Monitoring Committee
GEE: generalised estimating equation
Glau-QoL: Glaucoma Quality of Life Questionnaire
GLMM: generalised linear mixed models
GOAT: Glaucoma Outcome Assessment Tool
GITS: Glaucoma Initial Treatment Study
GPA: Guided Progression Analysis
HeNe: helium-neon laser
HREC: Human Research Ethics Committee
HRQoL: Health-Related Quality of Life
IQR: interquartile range
IRT: item response theory
ITT: intention to treat
IOP: intraocular pressure
MD: mean deviation
NHMRC: National Health and Medical Research Council
NICE: National Institute for Health and Care Excellence
NFLD: nerve fibre layer defects
OSD: ocular surface disease
ODOC: Optic Disc Outcomes Committee
OR: odds ratio
POAG: primary open angle glaucoma
QALY: quality-adjusted life year
QoL: quality of life
RCT: randomised controlled trial
SAE: serious adverse event
SD: standard deviation
SLT: selective laser trabeculoplasty
VFOC: Visual Field Outcomes Committee
WHO: World Health Organisation
XFG: exfoliative glaucoma
